# Description of first nursery area for a pygmy devil ray species (*Mobula munkiana*) in the Gulf of California, Mexico

**DOI:** 10.1038/s41598-020-80506-8

**Published:** 2021-01-08

**Authors:** Marta D. Palacios, Edgar M. Hoyos-Padilla, Abel Trejo-Ramírez, Donald A. Croll, Felipe Galván-Magaña, Kelly M. Zilliacus, John B. O’Sullivan, James T. Ketchum, Rogelio González-Armas

**Affiliations:** 1 Centro Interdisciplinario de Ciencias Marinas, Instituto Politécnico Nacional, 23096 La Paz, Mexico; 2 Pelagios Kakunjá A.C., 23060 La Paz, Mexico; 3 Ecology and Evolutionary Biology Department, University of California Santa Cruz, Santa Cruz, 95060 USA; 4 Fins Attached Marine Research and Conservation, Colorado Springs, 80908 USA; 5 Monterey Bay Aquarium, Monterey, CA 93940 USA; 6 Centro de Investigaciones Biológicas del Noroeste (CIBNOR), La Paz, 23096 México

**Keywords:** Animal behaviour, Ecology, Behavioural ecology, Conservation biology

## Abstract

Munk’s pygmy devil rays (*Mobula munkiana*) are medium-size, zooplanktivorous filter feeding, elasmobranchs characterized by aggregative behavior, low fecundity and delayed reproduction. These traits make them susceptible to targeted and by-catch fisheries and are listed as Vulnerable on the IUCN Red List. Multiple studies have examined fisheries impacts, but nursery areas or foraging neonate and juvenile concentrations have not been examined. This study describes the first nursery area for *M. munkiana* at Espiritu Santo Archipelago, Mexico. We examined spatial use of a shallow bay during 22 consecutive months in relation to environmental patterns using traditional tagging (n = 95) and acoustic telemetry (n = 7). Neonates and juveniles comprised 84% of tagged individuals and their residency index was significantly greater inside than outside the bay; spending a maximum of 145 consecutive days within the bay. Observations of near-term pregnant females, mating behavior, and neonates indicate an April to June pupping period. Anecdotal photograph review indicated that the nursery area is used by neonates and juveniles across years. These findings confirm, for the first time, the existence of nursery areas for Munk’s pygmy devil rays and the potential importance of shallow bays during early life stages for the conservation of this species.

## Introduction

Nursery areas have been shown to be important for many elasmobranch species^1,2^. These discrete areas have biotic and abiotic features that can be important for pupping and for enhancing the survival of neonates, and juveniles^2^. For an area to be considered an elasmobranch nursery, it must follow at least three criteria: (1) neonates, and juveniles are more commonly encountered within the area compared to adjacent areas, (2) individuals tend to remain or return to the area over weeks or months, and (3) the area is used in a similar manner repeatedly across years^3,4^.

While many studies have identified the importance of nursery areas for sharks^3,5,6^ little is known about nursery areas for batoids^7–9^. Indeed, only three important juvenile habitats for manta rays have been identified in the Gulf of Mexico^9,10^, in Florida^11^ (*Mobula birostris* and *Mobula.* cf. *birostris* for both areas), and in Indonesia^12^ (*Mobula alfredi*). In addition, a potential pupping ground for *Mobula mobular* in the Northern Gulf of California^13^, has been suggested, but more research is needed to confirm.

Mobulids (manta and devil rays) are planktivorous filter feeders with vulnerable life histories^14,15^ that include the lowest fecundity of all elasmobranchs (one pup per litter)^16,17^, and delayed, aplacental viviparous matrotrophic reproduction cycles of 1–3 years^18–21^. Such low reproductive rates make mobulids extremely vulnerable to anthropogenic impacts including targeted small-scale fisheries^18,22,23^ and bycatch in small- and large-scale fisheries^22,24^. As a result, all mobulid species are IUCN Red list, Endangered or Vulnerable^25^, with all species experiencing population declines^26,27^.

Pygmy devil rays (5 of the 10 mobulid species)^28^, include the smaller species reaching < 1.3 m disc width as adults with more restricted distribution than the larger mobulid species^15^. Munk’s pygmy devil ray (*Mobula munkiana*) is endemic to the Eastern Pacific, found in neritic and coastal habitats that extend from the Gulf of California, Mexico to Peru^29^. In the Gulf of California, *M. munkiana* feed predominantly upon Mysidacea spp. with the euphausiid, *Nyctiphanes simplex,* as a second prey item^18,30^.

While size at birth remains unknown, estimations and comparisons with other pygmy devils rays indicate that disc width could range from 35^18^ to 42.3 cm^31^, and reach up to 112 cm as an adult^32^. *Mobula munkiana* is particularly known for its social behavior^18^, often congregating in large aggregations of thousands of individuals, presumedly for mating purposes^15^. *M. munkiana* is currently classified as “Vulnerable” on the IUCN Red List of Threatened Species^29^. While the species is nationally protected in Mexican waters under the NOM-029-PESC-2006 and NOM-059-SEMARNAT-2010 regulations, illegal targeted fishing still exists in several areas in the Gulf of California^33^.

**Figure 1:**
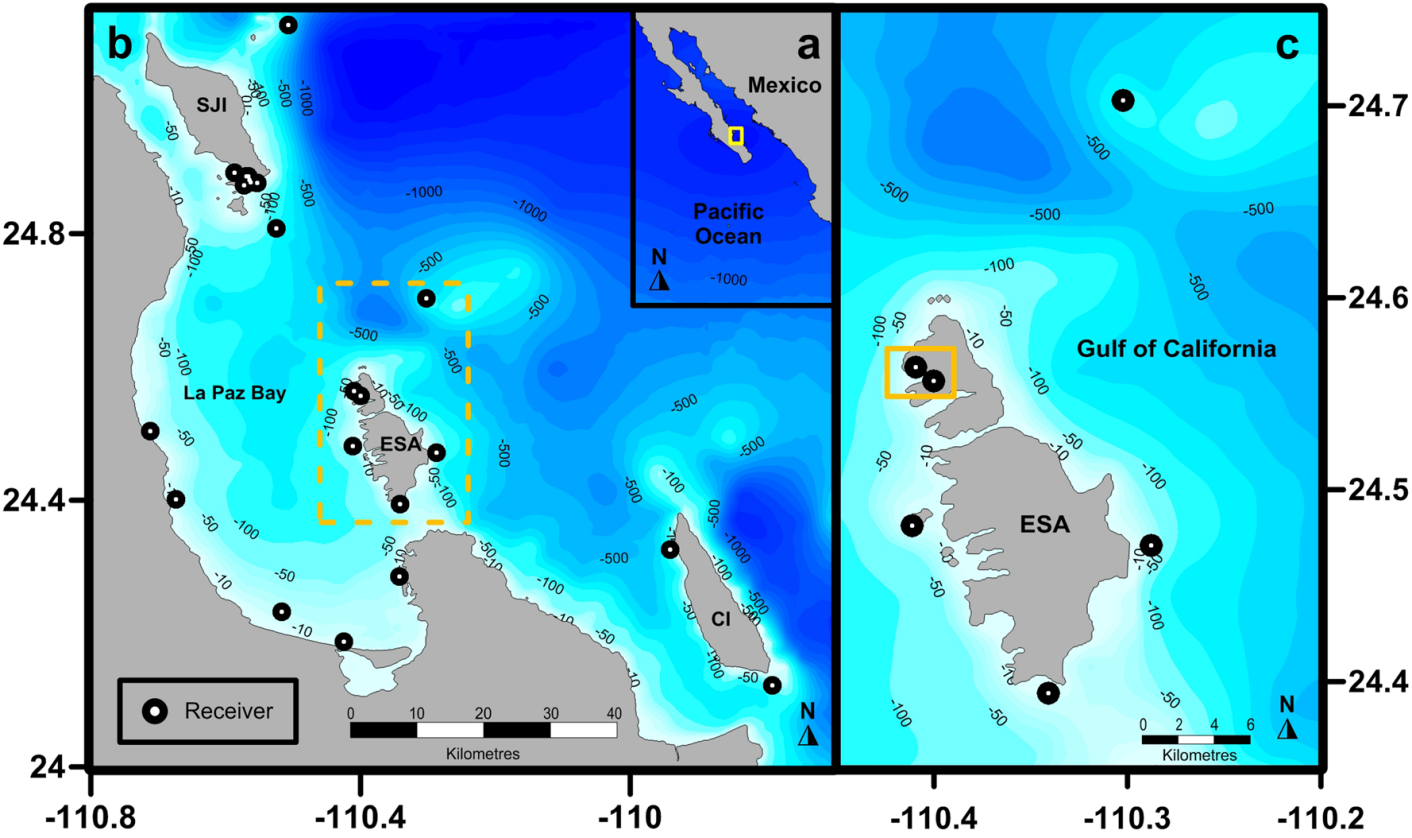
(**a**) Mexican Pacific and Baja California Peninsula. The yellow square located on the southwestern portion of Gulf of California outlines the location of (**b**) La Paz Bay and the surrounding islands of San Jose (SJI), Cerralvo (CI), and the Espiritu Santo Archipelago (ESA) outlined in yellow dashed square. The receiver locations (n = 21) are indicated with black–white dots. (**c**) *Mobula munkiana* early life stage individuals aggregate in the shallow bay of Ensenada Grande outlined with a yellow square. The map was created using Surface Mapping System (Golden Software, Inc., 1993–2012, https://www.goldensoftware.com/products/surfer) and the coastline data was extracted from GEODAS-NG (National Geophysical Data Center, 2000).

When *M. munkiana* was first described in the Southern Gulf of California, segregation by size was described^18,34,35^, leading to the potential for differential habitat use between juvenile and adult stages. Since 2013, local fishermen and tour operators in the Southern Gulf of California, have known of a well-established aggregation of *M. munkiana* in Ensenada Grande, a shallow bay with sandy bottom seafloor, located on the northwest side of the Espiritu Santo Archipelago (Fig. 1). These anecdotal observations prompted us to examine whether pygmy mobulid rays utilize nursery areas for mating, pupping, and foraging of juveniles.

Here we report the reproductive seasons (mating and parturition) for adults, and residency linked to environmental factors of early life stages of *M. munkiana* in a shallow bay at the Espiritu Santo Archipelago, Mexico. We used a combination of nonlethal methodologies including traditional tagging, passive acoustic telemetry, and environmental monitoring (zooplankton biovolume and water temperature) to examine the spatial use and foraging ecology of early life history stages of *M. munkiana* and to determine if *M. munkiana* utilize the shallow bay as a nursery area.

## Results

### Conventional tagging

**Figure 2:**
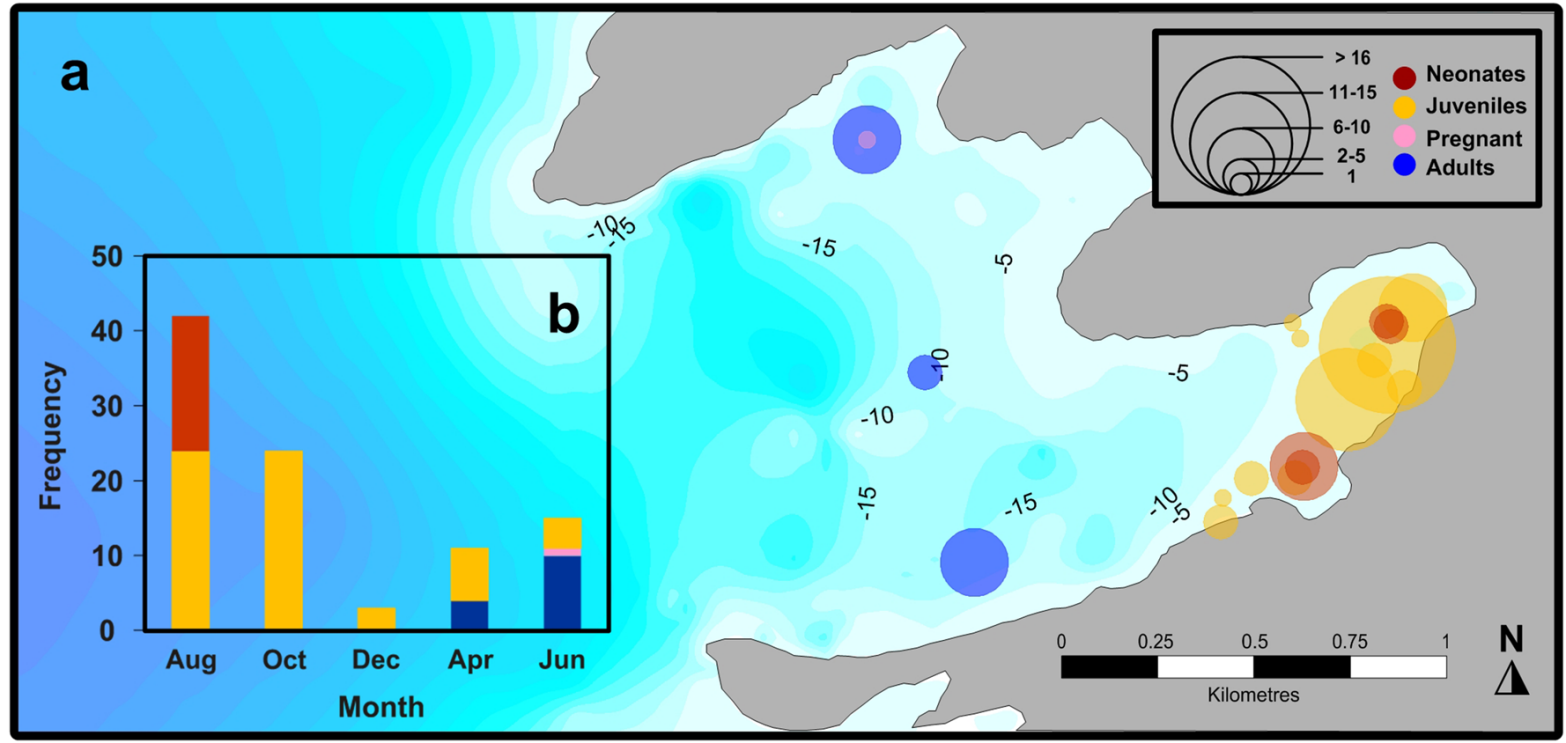
(**a**) Capture locations of *M. munkiana* between August 2017 and June 2018 at Ensenada Grande. Circle size indicates the number of individuals captured at each location by life stage. Numbers indicates the bathymetric lines. (**b**) Number of *M. munkiana* captured at Ensenada Grande per month and life stage following the same color code.

A total of 95 Munk’s pygmy devil rays were captured at Ensenada Grande from August 2017 to June 2018 during five capture periods (Supplementary Information Table S1). *Mobula munkiana* catches and life stage varied seasonally, with greater captures occurring during late summer and fall than during winter, spring, and early summer (Fig. 2). Disc width was not normally distributed (W_94_ = 0.925, *P* = 0.0004), and we found no significant difference in size by sex (W_93_ = 905, *P* = 0.18). Juveniles (65%, n = 62) and neonates (19%, n = 18) dominated the sampled population with a 1:1 sex ratio (X^2^ = 0.05, *P* = 0.8) with 39 females and 41 males.

Neonates (n = 18) were identified by the presence of the umbilical scar on the ventral side below the gills (Supplementary Information Figure S1). Neonate size ranged from 49.5 to 56 cm disc width and were only captured inside Ensenada Grande during August, at depths between 2 and 5 m. Juveniles (n = 62) ranged from 49 to 85 cm disc width and were captured during all sampling months at Ensenada Grande. Neonates and juveniles were only caught with individuals of the same life stage, indicating size segregation of the schools. All neonate and juvenile males had undeveloped claspers without calcification or rotation (Supplementary Information Figure S1), while neonate and juvenile females showed no evidence of mating scars and the state of the cloaca was not distended.

Adults (15%, n = 14) and pregnant females (1%, n = 1) were only captured during spring and early summer (April and June) at > 15 m depth in Ensenada Grande. The adults (n = 4) captured in April 2018 were females with swollen distended cloaca evidenced with a reddish coloration indicating possible recent mating or parturition (Supplementary Information Figure S1) as it has been interpreted in other elasmobranch species^36,37^.

During June 2018, we captured a group of adults composed of one female and four males displaying courtship behavior at the surface (initiation and endurance) as described for *Mobula alfredi* and *M. birostris*^38^. All four males had developed claspers with sperm. Courtship behavior was also observed during April 2018, but those animals were not captured. A female in an advanced state of pregnancy was captured at Ensenada Grande during June 2018 showing distended abdominal region on both the dorsal and ventral surface (Supplementary Information Figure S1). Pregnancy was confirmed on another individual with the same characteristics captured at Espiritu Santo Archipelago in April 2018 using ultrasound techniques, with a single and well-developed term-embryo present (Ramírez-Macías unpub. data). This corroborated the estimation of the litter size of a single pup for *M. munkiana*^39^ and other mobulid species^14,40^.

During this study we had seven recaptures (6.23%) of six individuals, four juveniles and two neonates. The straight-line capture/recapture distance for all recaptured devil rays was between 0.1 and 0.5 km, with recapture durations ranging from 1 day to 8 months from initial capture.

### Acoustic telemetry

#### Detection summary

**Figure 3:**
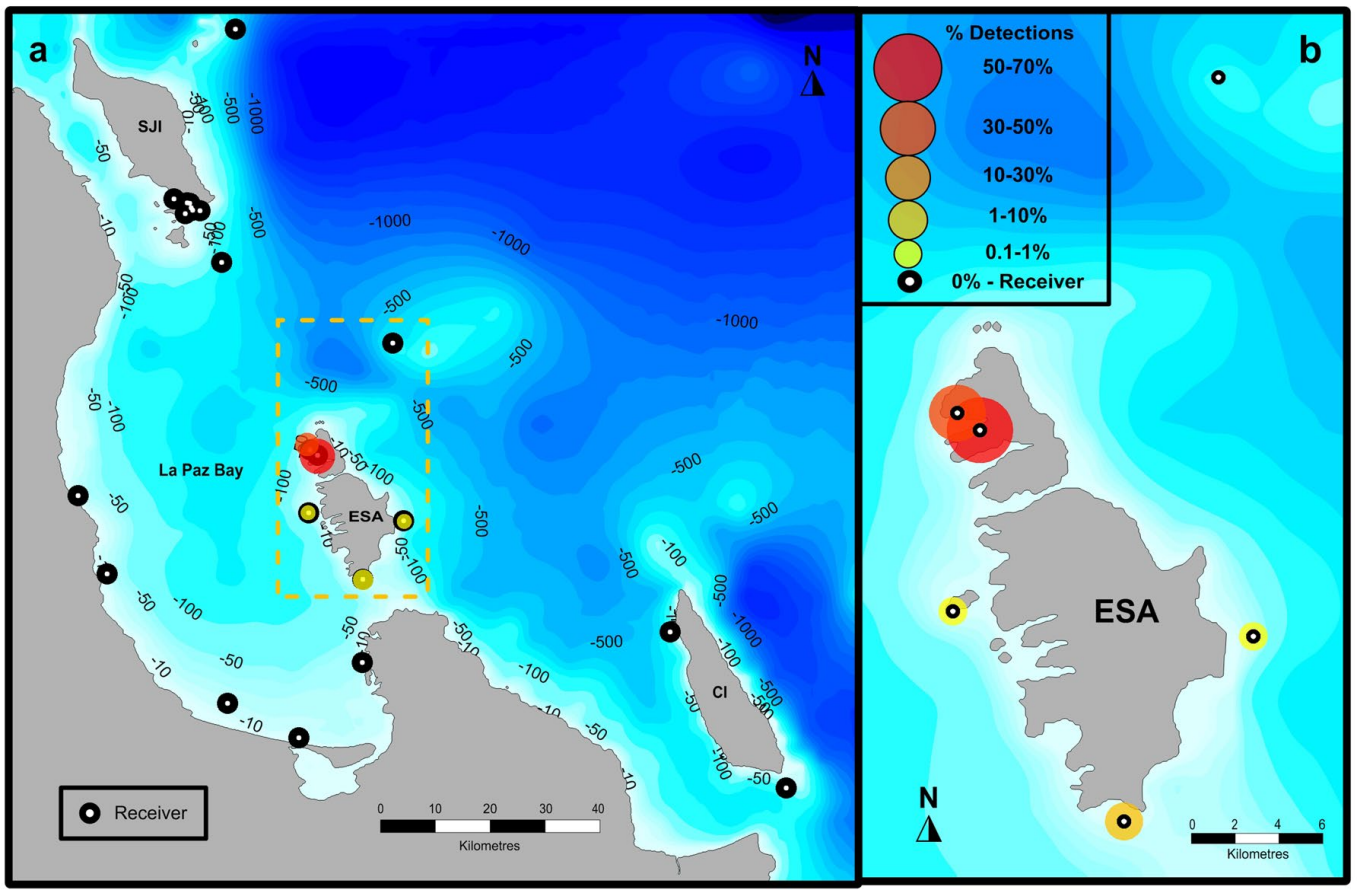
(**a**) *Mobula munkiana* detection map between August 2017 and May 2019 at La Paz Bay and the surrounding islands of San Jose (SJI), Cerralvo (CI), and (**b**) the Espiritu Santo Archipelago (ESA). The proportion (%) is indicated by circle size and color for each receiver described in the legend.

All seven acoustic tags deployed on *M. munkiana* (four neonates and three juveniles) (Table 1) were recorded by at least two receivers around the Espiritu Santo Archipelago. We recorded 38,275 detections for all individuals at five of the six receivers placed around Espiritu Santo Archipelago during the monitoring period (643 days) and no other detections were recorded on the rest of the acoustic array (n = 15) (La Paz Bay, Isla San Jose and Isla Cerralvo) (Fig. 3a).

**Table 1:** Summary of acoustic tag deployments on 4 neonates (1–4 ID) and 3 juveniles (5–7 ID) of *M. munkiana* at Ensenada Grande (EG), Espiritu Santo Archipelago in 2017. Dates are given as d/mo/yr. DW, disc width; no. det, number of detections; det, detections.

Mobula ID	Sex	DW (cm)	Deployment date	Last detection	Total no. det	Total track days	Total det. days	Residency index EG (%)	Max no. of consecutive days det at EG
1	F	50	02/08/17	08/01/18	15,432	151	150	99	145
2	M	50	02/08/17	21/10/18	4777	437	50	11	46
3	M	52	02/08/17	09/01/19	5582	498	139	26	26
4	F	55	02/08/17	16/04/19	9011	614	129	20	70
5	F	72	01/08/17	22/03/19	208	537	16	1	1
6	M	72	01/08/17	29/04/19	3248	631	173	26	17
7	F	75	02/08/17	01/03/18	27	183	9	4	3

Females accounted for 64.5% of detections (two neonates with 63.9% and two juveniles with 0.6% of total detections), while males accounted for 35.5% of detections (two neonates with 27% and one juvenile with 8.5% of total detections).

#### Residency

Overall residency indices for Espiritu Santo Archipelago-tagged individuals ranged from 1 to 99% (27 ± 33%, mean ± SD). The tracking duration for individual *M. munkiana* ranged from 151 to 631 days (435 ± 195 days, mean ± SD). Detections on consecutive days were found in receivers both within (maximum 145 consecutive days) and outside Ensenada Grande (maximum of three consecutive days). Neonates were present at Ensenada Grande during 26 to 145 successive days while juveniles were present from 1 to 17 successive days. There were no significant differences in the residency index between sexes (W_6_ = 4, *P* = 0.63), maturity stages (W_6_ = 2, *P* = 0.23) or sizes (S = 88.59, *P* = 0.17).

#### Habitat preference and spatial movements

Areas of high activity as determined by the number of detections of tagged Munk’s pygmy devil rays were in coastal waters inside Ensenada Grande where 98.6% of the validated receiver detections were registered (Fig. 3b). The other receivers around the Espiritu Santo Archipelago were categorized as offshore and accounted for just 1.4% of the detections, while no detections were registered in the remainder of the receiver array (Fig. 3a).

As a result, the Ensenada Grande receivers had a statistically greater residency index compared to other receivers placed around the Espiritu Santo Archipelago (W_13_ = 44, *P* = 0.01). Individuals moved throughout the Espiritu Santo Archipelago with a travelling minimum linear dispersal distance of 18.5 ± 7.6 km (mean ± SD) and a maximum of 21.4 km based on detections around the archipelago. One single individual (neonate, 50 cm disc width) was never detected outside of Ensenada Grande, and had a minimum linear dispersal distance of only 1.22 km.

#### Seasonality

**Figure 4:**
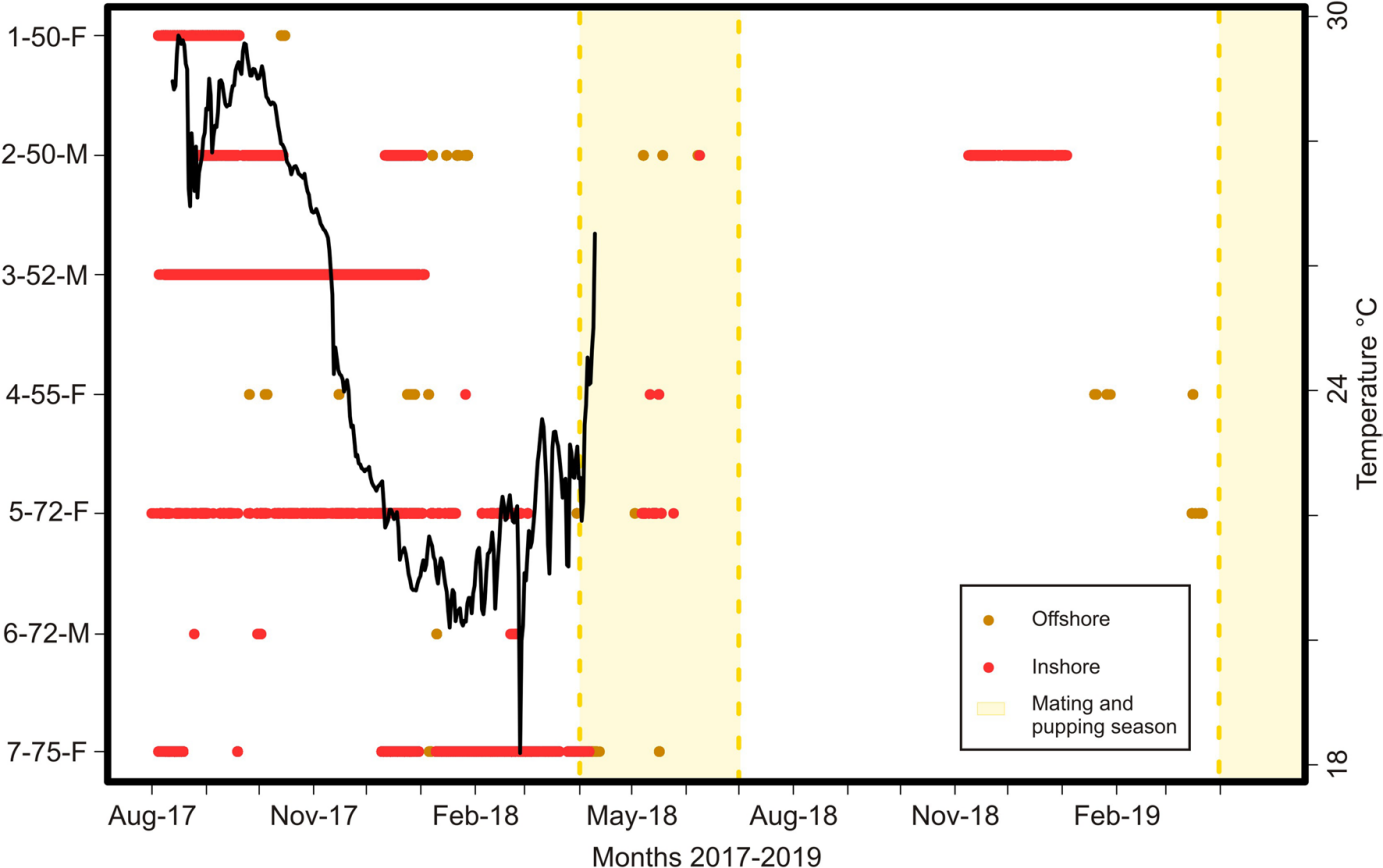
*Mobula munkiana* (n = 7) detections recorded at Espiritu Santo Archipelago between August 2017 and May 2019. Left axis specified code of the animal tag–disc width in centimeters–Sex (F: female; M: male). Ensenada Grande receivers are indicated in red (Inshore) and the rest of the Espiritu Santo Archipelago array is in orange (Offshore). Black line represents the temperature at Ensenada Grande from August 2017 to April 2018. Months of mating and pupping season are indicated in yellow.

Acoustic detections occurred at the Espiritu Santo Archipelago throughout the year for most devil rays, with no statistically significant differences in residency index between warm and cold seasons (W_53_ = 249, *P* = 0.07). The largest residency indices included September, October, November (warm season), and December (start of cold season) 2017 (Fig. 4). Detection rates for all tagged neonates and juveniles decreased during March and April when adults tend to be more frequent at Ensenada Grande and Espiritu Santo Archipelago. Larger juveniles also appear to recruit into the adult population sometime between April and June, supported by our field observation of a tagged (conventional tag) juvenile (≈ 85 cm disc width) swimming in the deeper part of Ensenada Grande (> 20 m) as part of a large school of *M. munkiana* adults.

#### Diel change

**Figure 5:**
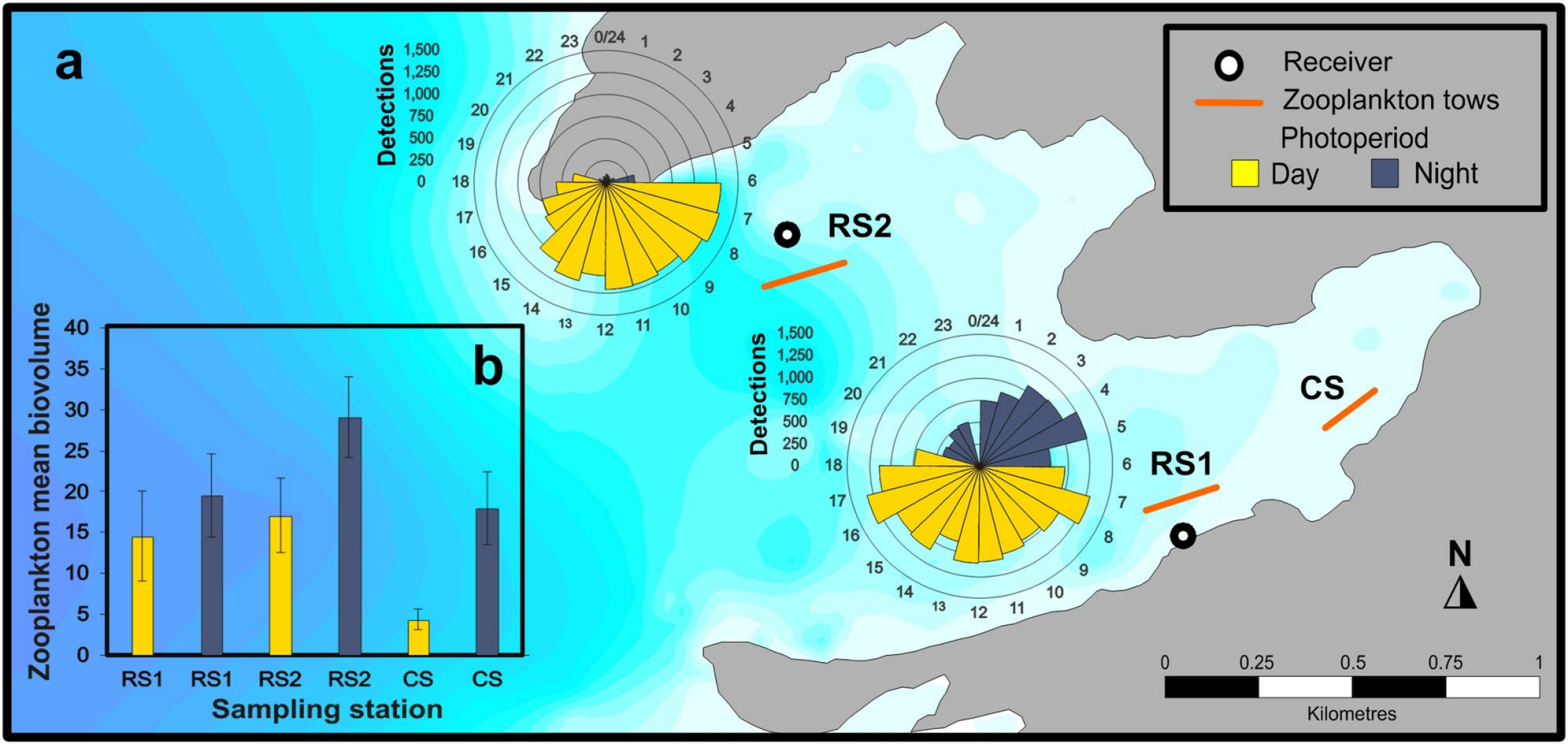
(**a**) Receiver locations (black–white dots: RS1 at 5 m depth and RS2 at 26 m depth) and zooplankton sampling stations (orange lines) locations at Ensenada Grande. Circular plots of detections per hour of acoustic tagged *M. munkiana* (n = 7) at each receiver. (**b**) Zooplankton mean biovolume (mL 100 m^−3^) and standard error at the three sampling stations collected at day and night during the study period.

All detections at Ensenada Grande showed that the spatial distribution of Munk’s pygmy devil rays varied by time of the day (Fig. 5a). Tagged *M. munkiana* were detected by the shallow receiver, RS1 (5 m depth) during all hours, but detections were significantly more frequent during daytime (U = 359.6, *P* < 0.05). We found three peaks in detections: between 0400–0500 h (nighttime), 0700–0800 h (daytime) and 1600–1700 h (daytime). We also found significantly greater detections during the daytime at the receiver placed in a deeper area within Ensenada Grande, RS2 (26 m depth) (U = 359.39, *P* < 0.05) with almost no detections during nighttime when *M. munkiana* appear to move to shallower areas.

### Environmental factors

#### Temperature

Sea water temperature from Ensenada Grande was recorded from August 2017 until April 2018. Temperature values followed seasonal patterns previously described^41^ with maximum temperatures from June to November (24.1–29.6 °C) and minimum values from December to May (18.1–26.5 °C). We found a statistically significant correlation between water temperature and the mean monthly residency index of tagged *M. munkiana* at Ensenada Grande (S = 2.496^e+09^, *P* < 0.0001, rho = 0.643). Detections of tagged individuals were consistently greater (up to 145 days of consecutive detections) between August to April of the first year of the study (2017–2018) when water temperature ranged from 18.8 to 29.6 °C, suggesting that they may range less widely during those months of the year. About 77% of the detections in Ensenada Grande occurred when water temperature ranged 25.5–29.6 °C (total range 18.1 to 29.6 °C) (Fig. 4).

#### Zooplankton

Zooplankton was primarily composed of major taxonomic groups of holoplankton (Copepoda, Cladocera, Euphausiids, Chaetognatha, Mysidacea and Decapoda).

Zooplankton biovolume was significantly greater during the night compared to day (W_126_ = 1175, *P* = 0.0001549) (Fig. 5b) across all sampling months, with a peak value of 36.27 ± 8.25 mL 100 m^−3^ (mean ± SEM) during nighttime samples in December. We found a significantly greater mean zooplankton biovolume during the cold season (December to May) (W_126_ = 2454.5, *P* = 0.027) as well as between months (Kruskal–Wallis X^2^ = 23.1, df = 5, *P* = 0.0003), with maximum zooplankton biovolume values observed during December (31.12 ± 4.98 mL 100 m^−3^, mean ± SEM) and lowest values in June (10.91 ± 1.64 mL 100 m^−3^, mean ± SEM). We also found significant differences of zooplankton biovolume across our three sampling stations inside Ensenada Grande (Kruskal–Wallis X^2^ = 13.478, df = 2, *P* = 0.00118; Dunn test, *P* < 0.05). The deeper station had significantly greater nighttime zooplankton biovolume (29.13 ± 4.89 mL 100 m^−3^, mean ± SEM) even though we mainly detected devil rays during night hours at the shallower station where mean zooplankton biovolume values were lower (19.55 ± 5.08 mL 100 m^−3^, mean ± SEM). Nevertheless, mean monthly residency index and the zooplankton biovolume within Ensenada Grande were significantly positively correlated (S = 221,340, *P* = 5.046^e−05^, rho = 0.3516104).

## Discussion and conclusions

**Figure 6:**
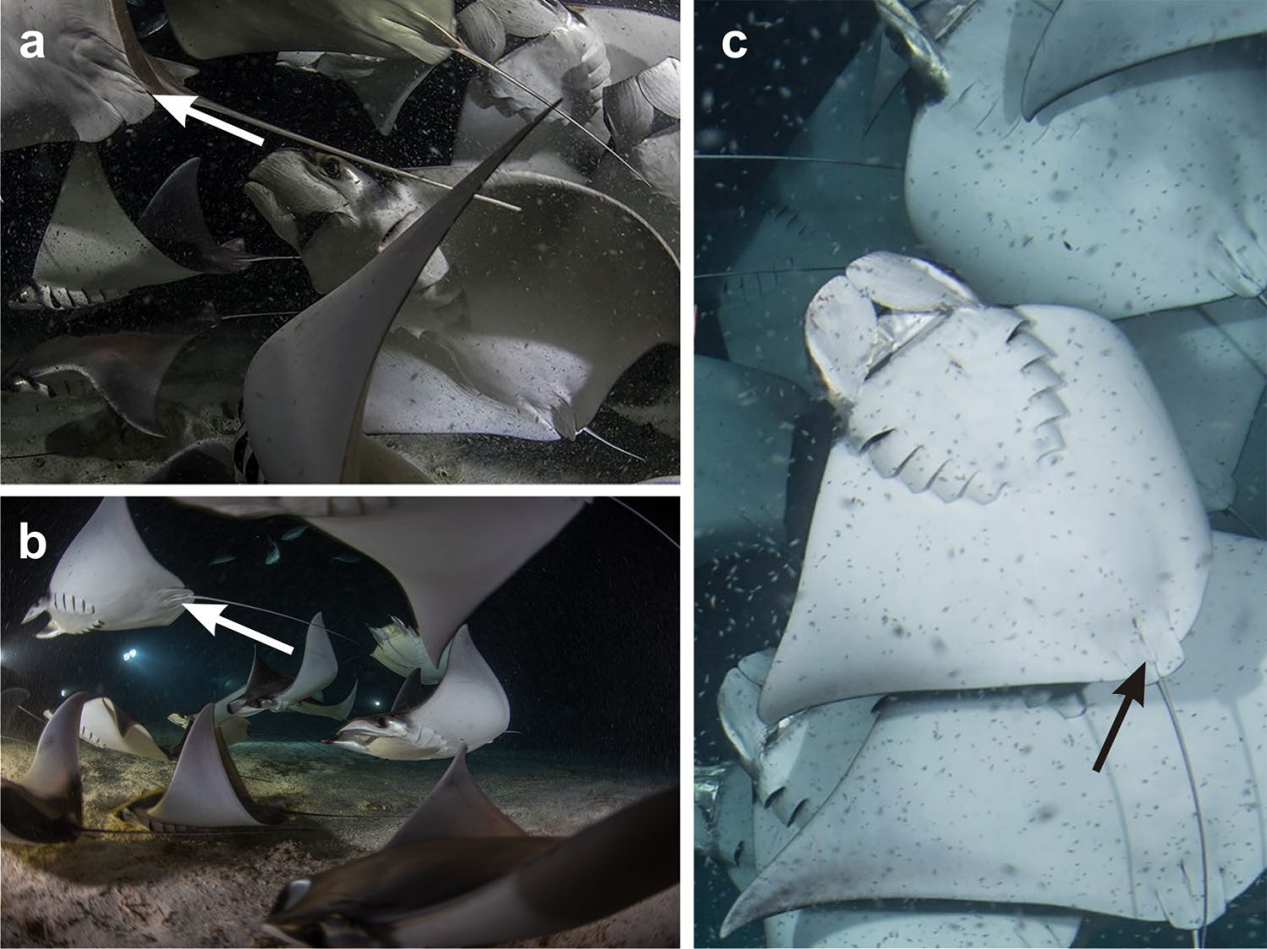
Juvenile males *M. munkiana* with undeveloped claspers (indicated by arrows) at Ensenada Grande during recreational dives in (**a**) November 2013, (**b**) October 2014 and (**c**) October 2016. Images copyright: (**a**), (**b**) Erick Higuera and (**c**) Luke Inman.

Our results indicate that *M. munkiana* utilize nursery areas following the definition proposed for elasmobranch nursery areas. The Ensenada Grande bay of the Espiritu Santo Archipelago can be considered a nursery area for *M. munkiana* following the three criteria:

1. Neonate and juvenile rays are more commonly encountered in Ensenada Grande than in other areas due to their high relative abundance, 84% (n = 80) compared with other studies^18,32^ where proportions for neonates (8.3%, n = 2) and juveniles (15%, n = 22) captured were much lower in adjacent areas.
2. Neonates and juveniles exhibited greater residency indices in Ensenada Grande, being detected almost daily for up to 7 of the 22 months monitoring period in the bay. Individuals resided inside this inshore area from 1 to 145 consecutive days. Moreover, recapture data from traditional tagging demonstrated a site fidelity of 2 to 8 months inside Ensenada Grande for neonates and juveniles.
3. *Mobula munkiana* neonates and juveniles use Ensenada Grande as a nursery area across multiple years. Using anecdotal professional photographs from 2013 to 2016 (Fig. 6), there is evidence that since ecotourism activities started, sightings of *M. munkiana*, including juveniles and neonates, are common each year from September to December.

Furthermore, our results provide compelling evidence that *M. munkiana* use Ensenada Grande as a primary nursery area^42^ due to the presence of neonates and near-term pregnant females, and as a secondary nursery area^42^ due to the presence of juveniles (non-newborn). Therefore, overlapping primary and secondary nursery areas for pygmy devil ray species occurs, similar to that observed for other elasmobranch species^3^.

The Southern Gulf of California was previously thought to be a wintering ground for *M. munkiana*, with them disappearing from the region during the warmer season for mating and pupping^18^. However, we instead propose the use of shallow bays adjacent to high secondary production, such as Ensenada Grande, as a nursery area where neonates and juveniles likely remain throughout the year. We suggest that there are likely other similar, yet undiscovered, nursery areas elsewhere in the Gulf of California and Eastern Pacific for this species. We found that early life stage *M. munkiana* exhibited a higher residency index during warmer water temperatures. This warm temperature residency may provide an ecological advantage by accelerating the metabolic rates and thus growth of juveniles and thereby reducing the duration of these vulnerable life-history stages^3,43^. The habitat preference of one of its main prey Mysidacea spp., in shallower parts of the neritic zone^44^, combined with protection of *M. munkiana* early life stages from large predators, could partially explain the higher detection rate recorded at shallower receivers. As a result, there appears to be an advantage for *M. munkiana* neonates and juveniles to behave as residents with a high fidelity to shallow-coastal habitats in contrast to adults which range widely in oceanic waters.

We observed a clear ontogenetic spatio-temporal segregation among neonates, juveniles, and adults since these different life stages were all caught during different seasons and areas within Ensenada Grande. Size segregation appears to be a common feature for this and other species of mobulids^18,45^. Although sex segregation has been reported in the southern part of the Gulf of California across different years for primarily adult *M. munkiana*^18,32,39^, we found a 1:1 sex ratio for neonates and juveniles, a typical feature in elasmobranch nursery areas^1,46^. This suggests that *M. munkiana* does not segregate by sex during early stages but perhaps may initiate sex segregation when they reach sexual maturity.

Reproductive seasonality has been documented for several mobulid species^38,40^. Based on our information we suggest that the mating and pupping season for *M. munkiana* begins in April and ends in June when water temperatures range between 18 and 29 °C. Parturition for *M. munkiana* in La Paz Bay has been previously reported between May and June^39^, however based on our observations of near term pregnant females in April and June, females with signs of possible parturition in April, and the neonate sizes in August we believe that an extended pupping season is feasible.

This time frame coincides with a transition around June from the cold season when the euphausiid, *N. simplex*, one of the two main *M. munkiana* prey items^30^, attains its maximum abundance and reproductive period in the Gulf of California^47–49^. A gestation period of 10 to 12 months has been reported for another pygmy devil ray, *Mobula eregoodootenkee*^31^ (originally cited as *M. kuhlii* cf. *eregoodootenkee*) with very similar body size^15,22^, therefore is very likely that Munk’s pygmy devil ray gestation period is the same. Indeed, we observed courtship and pregnancy in the same area and time period in La Paz Bay. The timing of parturition and mating are further supported by observations of *M. alfredi* in captivity^50^ and wild individuals^38^.

This is the first description of a pygmy devil ray nursery area and the habitat used by neonates and juveniles within it. Individuals of early life stages displayed a high level of residency to the area, more correlated to warmer temperatures than to zooplankton abundance. Nursery and mating grounds for devil rays are highly likely to overlap in temporal and geographic space. Ultimately, since devil rays have the lowest fecundity of all elasmobranchs^17^, this information may be useful in the design of spatial and temporal management strategies to mitigate bycatch in artisanal fishing and to regulate ecotourism activities not only within the Southern Gulf of California, but elsewhere throughout their range. In addition, the information presented here will be useful in identifying nursery areas for other devil ray species world-wide.

## Methods

### Study area

The Espiritu Santo Archipelago is located in the south west region of the Gulf of California and is the eastern limit of La Paz Bay (Fig. 1). The Archipelago was declared a Marine National Park in 2007, only allowing artisanal fisheries and ecotourism activities in some restricted areas. The bathymetry of the eastern Espiritu Santo Archipelago is characterized by steep terracing, with water over 100 m occurring just a few meters from the shore, particularly off the eastern side of the archipelago. Our main study area at Ensenada Grande is located on the western coast of the Espiritu Santo Archipelago and is comprised of several sandy bottom embayment’s (< 40 m depth) (Fig. 1c). Productivity of the Espiritu Santo Archipelago is influenced by the monsoonal wind pattern of the Gulf of California with northwesterly winds that cause strong upwelling events during the cold season (December to May), with primary production rates ranging between 1.16 and 1.91 g C m^2 ^d^−1^^51^. Strong thermal stratification occurs during the warm season (June to November), when upwelling is weak along the east coast of Baja California peninsula^52^ with low primary production rates (0.39 to 0.49 g C m^2 ^d^−1^)^51^.

### Data collection

*Mobula munkiana*, were caught between August 2017 and June 2018 at Ensenada Grande during 5 capture trips. Individuals were captured with encircling surface cotton twine nets 150 m long, 15 m deep, with 25 cm mesh. Captured individuals were maintained in the water, allowing water to pass over their gills to reduce stress levels before transferring them into a holding tank onboard the boat. Individuals were sexed, measured (total length and disc width), and evaluated for mating scars on pectoral fins, cloacal state (females) and development state of claspers (males). Release was typically completed < 5 min after capture and all devil rays were released in good condition.

### Life stages description

*Mobula munkiana* maturity was classified in four states, according to estimates of their disc width size at maturity as either neonate (< 97 cm female or 98 cm male disc width with umbilical scars present), juvenile (< 97 cm female or 98 cm male disc width with no umbilical scar) and adult (> 97 cm female or 98 cm male disc width)^32^. Adult females with a noticeably distended abdominal region on both the dorsal and ventral surfaces were classified as likely pregnant females^20^.

### Conventional tagging

Individuals were tagged with conventional fish tags (FLOY TAG & Mfg., Inc.) in the dorsal part of the pectoral fin with a special applier for future identification purposes.

The data collected from captures and conventional tagging were used to characterize the overall size and demographic composition of the population captured in Ensenada Grande. A X^2^ test was used to test for skewed sex ratios in captured juveniles and neonates in Ensenada Grande.

Size data set did not meet the normality assumptions according to the Shapiro–Wilk test (n = 95, W = 0.92567, *P* = 4.446e−05), therefore a nonparametric Wilcoxon test was performed to compare disc width and sex distribution. Capture locations were plotted using Surface Mapping System (Golden Software, Inc., 1993–2012, https://www.goldensoftware.com/products/surfer) and the coastline data was extracted from GEODAS-NG (National Geophysical Data Center, 2000).

### Acoustic telemetry

*Mobula munkiana* were fitted with internal acoustic transmitters (Vemco Ltd. V13; n = 7) with an expected battery life of 991 days in August 2017 at Ensenada Grande. Transmitters were coated with a beeswax/paraffin wax mixture and internally placed by surgically inserting them into a 3 cm incision in the abdominal cavity. The incision was closed with synthetic surgical sutures. Transmitters operated at 69 kHz and were coded to pulse randomly once every 40–80 s allowing the simultaneous monitoring of multiple individuals without continuous signal overlap. Acoustic receivers (model VR2w and VR2Tx Vemco Ltd; n = 6) were moored at depths between 5 and 26 m at locations previously known to be frequented by Munk’s pygmy devil rays within the Espiritu Santo Archipelago as part of a larger receiver array (n = 21 receivers) installed within La Paz Bay, Cerralvo Island, and San Jose Island, providing a much greater coverage of our main Ensenada Grande study site and adjacent areas (Fig. 1). We tested acoustic array range and found a maximum detection range of 350 m for the receivers at the Espiritu Santo Archipelago. Receivers recorded the transmitter code, time, and date of tagged *M. munkiana* that swam within the detection range of the receivers. Movements of neonates and juveniles *M. munkiana* were monitored on the array between August 2017 and May 2019.

Receiver data in this network were downloaded and batteries are changed at least annually, and data were processed using the VUE Software (Vemco Inc., https://support.vemco.com/s/downloads). We filtered the data to include only detections with two or more consecutive detections as a means to avoid false positive detections that could arise from background noise^53^.

The distribution and residency of detections throughout the receiver array were visualized and analyzed using the package “VTrack” (https://CRAN.R-project.org/package=VTrack) in R (https://www.r-project.org/). A residency index^54^ for each individual captured in the Espiritu Santo Archipelago was calculated with the formula (1).

1 $$Residency\, Index (\%) = \frac{No.\, of\, days\, detected}{No.\, of\, days\, between\, first\, and\, last \, detection}$$

The sequential series of detections over time throughout the receiver array from the first detection to the last is referred to as the “track” for each individual.

Daily presence data were analyzed to determine the number of consecutive days that an individual was resident (continuous presence) at a location. Since the acoustic data set did not meet the normality assumptions according to the Shapiro–Wilk test (n = 7; W = 0.78852, *P* = 0.03148) a nonparametric Spearman correlation and Wilcoxon tests were carried out to determine whether residency indices differed significantly with disc width, sex, and maturity stage of tagged *M. munkiana*. Habitat preference was studied by grouping the acoustic receivers of Ensenada Grande (n = 2) as inside-bay receivers and the rest of the Espiritu Santo Archipelago acoustic array (n = 4) as offshore receivers. A Wilcoxon test was used to compare the residency index found inside-bay receivers versus offshore. Differences in residency between seasons was examined by comparing monthly residences of warm months (June to November) against cold months (December to May) using a Wilcoxon test. To quantify diel changes in the *M. munkiana* presence of Ensenada Grande we produced circular plots of the number of detections during daytime (0600–1900 h) versus nighttime (1900–0600 h); limits of diel times were determined using defined cutoffs for dawn and dusk for the Ensenada Grande location. We used Rao’s test to analyze the uniformity of the detections for the receivers inside Ensenada Grande. We calculated the minimum linear dispersal distance for each individual defined as the distance between the two furthest receivers at which an individual was ever detected using Surface Mapping System (Golden Software, Inc., 1993–2012, https://www.goldensoftware.com/products/surfer).

### Environmental factors

Water temperature data was collected every 2 h by a temperature logger (Onset HOBO Water Temperature, Pendant 64 k) deployed at Ensenada Grande at 13 m depth during 9 months from August 2017 to April 2018. Temperature records were averaged over each day of the study period and aligned with the acoustic detection data to examine temperature effects on mobulid presence/absence.

Zooplankton was sampled during day and night at three locations inside Ensenada Grande (Fig. 5a). A total of 125 zooplankton samples were collected from August 2017 to June 2018 (25 samples per monitored month). Zooplankton was collected during a three minute oblique tow with a 60 cm mouth diameter zooplankton net (300 μm mesh), equipped with a calibrated flow meter (G.O. 2030R) mounted in the mouth of the net to estimate the filtered seawater volume^55^. Samples were preserved with 4% formalin. Zooplankton biovolume (mL 100 m^−3^) was estimated for each sample using the displacement volume method^56^.

Temperature (n = 3466 W = 0.91839, *P* = 1.848e−05) and zooplankton biovolume (n = 128, W = 0.75869, *P* = 3.424e−11) data sets did not meet the normality assumptions according to the Shapiro–Wilk test respectively, therefore nonparametric Wilcoxon tests were used to compare seawater temperatures among seasons and zooplankton biovolume between day/night and between warm/cold seasons. We tested the correlation between the seawater temperature and zooplankton biovolume with the mean monthly residency index at Ensenada Grande using Spearman correlations. Kruskal Wallis non-parametric tests were used to compare the zooplankton biovolume among months and sampling stations and post-hoc Dunn test were used to determine which months and sampling stations significantly differed.

### Ethical approval

The methods were approved under the research permit PPF/DGOPA-133/17 issued by Comisión Nacional de Acuacultura y Pesca with authorization of Comisión Nacional de Áreas Naturales Protegidas. The tagging and surgical procedures followed the Institutional Animal Care and Use Committee of the University of California, Davis (IACUC, Protocol No. 16022).

## Supplementary Information

Supplementary Information

Supplementary Figure S1.

## Data Availability

The dataset of acoustic detections of *M. munkiana*, receiver range test, the Ensenada Grande temperature, and zooplankton measurements generated and analyzed during the current study are available from the corresponding author on reasonable request.
