# Supplementary material for: Description of first nursery area for a pygmy devil ray species (Mobula munkiana) in the Gulf of California, Mexico
Source: Sci Rep. 2021 Jan 8;11:132. doi: 10.1038/s41598-020-80506-8 (PMC7794486; doi:10.1038/s41598-020-80506-8)
Supplement: Supplementary file 1 — Supplementary Information [file 41598_2020_80506_MOESM1_ESM.docx]

**Supplementary Information**

**Description of first nursery area for a pygmy devil ray species (*Mobula munkiana*) in the Gulf of California, Mexico.**

**Marta D. Palacios**^1,2+^**, Edgar M. Hoyos-Padilla**^2,4^, **Abel Trejo-Ramírez**^2+^**, Donald A. Croll**^3^**, Felipe Galván-Magaña**^1^**, Kelly M. Zilliacus**^3^**, John B. O’Sullivan**^5^**, James T. Ketchum**^2,6^, **Rogelio González-Armas**^1*^

1Instituto Politécnico Nacional, Centro Interdisciplinario de Ciencias Marinas, La Paz, 23096, Mexico

2Pelagios Kakunjá A.C., La Paz, 23060, Mexico

3University of California Santa Cruz, Ecology and Evolutionary Biology Department, Santa Cruz, 95060, USA

4Fins Attached Marine Research and Conservation, Colorado Springs, 80908, USA

5Monterey Bay Aquarium, Monterey, California, 93940, USA

6Centro de Investigaciones Biológicas del Noroeste (CIBNOR), La Paz, 23096, México

[*rogelio.glez.armas59@gmail.com](mailto:*rogelio.glez.armas59@gmail.com)

**Figure S1.** **(a)** Neonate Munk’s devil ray with the umbilical cord scar and **(b)** juvenile male with undeveloped claspers (indicated by arrows) captured at Ensenada Grande in August 2017. **(c)** Adult female with swollen distended cloaca evidenced with a reddish coloration at Ensenada Grande in April 2018. **(d)** Pregnant female showing distended abdominal region at Ensenada Grande in June 2018.

**Table S1.** Summary of traditional tagged individuals (n= 95) at Ensenada Grande from August 2017 to June 2018. Capture Date is d/mo/yr; C/R: Capture / Recapture; Sex: M (Male) / F (Female); DW: disc width; TL: Total length. Acoustically tagged individuals are shown in yellow.

| Latitude | Longitude | Capture  Date | Mobula ID | C/R | Sex | Maturity Stage | DW (cm) | TL (cm) | Observations |
| --- | --- | --- | --- | --- | --- | --- | --- | --- | --- |
| 24.5616 | -110.398 | 01/08/2017 | 426 | C | M | Juvenile | 81.0 | 47.5 |  |
| 24.56 | -110.395 | 01/08/2017 | 437 | C | F | Juvenile | 76.0 | 45.0 |  |
| 24.56 | -110.395 | 01/08/2017 | 355 | C | M | Juvenile | 75.0 | 40.0 |  |
| 24.56 | -110.395 | 01/08/2017 | 1 | C | M | Juvenile | 72.0 | 40.0 |  |
| 24.56 | -110.395 | 01/08/2017 | 2 | C | F | Juvenile | 72.0 | 40.0 |  |
| 24.5581 | -110.397 | 02/08/2017 | 356 | C | M | Neonate | 56.0 | 31.0 | Umbilical scars |
| 24.5581 | -110.397 | 02/08/2017 | 3 | C | M | Neonate | 52.0 | 29.0 | Umbilical scars |
| 24.5581 | -110.397 | 02/08/2017 | 408 | C | F | Neonate | 52.0 | 30.0 | Umbilical scars |
| 24.5581 | -110.397 | 02/08/2017 | 446 | C | M | Neonate | 52.0 | 30.0 | Umbilical scars |
| 24.5581 | -110.397 | 02/08/2017 | 445 | C | M | Neonate | 51.0 | 29.0 | Umbilical scars |
| 24.5581 | -110.397 | 02/08/2017 | 4 | C | F | Neonate | 50.0 | 29.0 | Umbilical scars |
| 24.5581 | -110.397 | 02/08/2017 | 5 | C | M | Neonate | 50.0 | 31.0 | Umbilical scars |
| 24.5581 | -110.397 | 02/08/2017 | 6 | C | F | Neonate | 55.0 | 33.0 | Umbilical scars |
| 24.5581 | -110.397 | 02/08/2017 | 350 | C | F | Neonate | 54.0 | 32.0 | Umbilical scars |
| 24.5581 | -110.397 | 02/08/2017 | 429 | C | F | Neonate | 51.0 | 29.0 | Umbilical scars |
| 24.561 | -110.395 | 02/08/2017 | 438 | C | M | Juvenile | 79.0 | 45.0 |  |
| 24.561 | -110.395 | 02/08/2017 | 436 | C | F | Juvenile | 78.0 | 44.0 |  |
| 24.561 | -110.395 | 02/08/2017 | 358 | C | M | Juvenile | 78.0 | 46.0 |  |
| 24.561 | -110.395 | 02/08/2017 | 435 | C | M | Juvenile | 77.5 | 45.0 |  |
| 24.561 | -110.395 | 02/08/2017 | 373 | C | M | Juvenile | 77.0 | 43.0 |  |
| 24.561 | -110.395 | 02/08/2017 | 434 | C | M | Juvenile | 76.0 | 43.0 |  |
| 24.561 | -110.395 | 02/08/2017 | 7 | C | F | Juvenile | 75.0 | 42.0 |  |
| 24.561 | -110.395 | 02/08/2017 | 363 | C | M | Juvenile | 75.0 | 42.0 |  |
| 24.561 | -110.395 | 02/08/2017 | 369 | C | F | Juvenile | 75.0 | 41.0 |  |
| 24.561 | -110.395 | 02/08/2017 | 449 | C | F | Juvenile | 75.0 | 43.5 |  |
| 24.561 | -110.395 | 02/08/2017 | 425 | C | M | Juvenile | 74.0 | 41.0 |  |
| 24.561 | -110.395 | 02/08/2017 | 444 | C | M | Juvenile | 74.0 | 38.0 |  |
| 24.561 | -110.395 | 02/08/2017 | 443 | C | M | Juvenile | 73.0 | 41.0 |  |
| 24.561 | -110.395 | 02/08/2017 | 431 | C | F | Juvenile | 72.0 | 44.0 |  |
| 24.561 | -110.395 | 02/08/2017 | 353 | C | F | Juvenile | 72.0 | 43.0 |  |
| 24.561 | -110.395 | 02/08/2017 | 428 | C | M | Juvenile | 71.5 | 42.0 |  |
| 24.561 | -110.395 | 02/08/2017 | 346 | C | M | Juvenile | 70.5 | 41.0 |  |
| 24.561 | -110.395 | 02/08/2017 | 448 | C | F | Juvenile | 70.0 | 40.0 |  |
| 24.561 | -110.395 | 02/08/2017 | 370 | C | F | Juvenile | 69.0 | 42.0 |  |
| 24.5615 | -110.395 | 03/08/2017 | 366 | C | F | Neonate | 55.0 | 31.0 | Umbilical scars |
| 24.5615 | -110.395 | 03/08/2017 | 397 | C | F | Neonate | 54.0 | 31.0 | Umbilical scars |
| 24.5615 | -110.395 | 03/08/2017 | 8 | C | F | Neonate | 53.0 | 29.5 | Umbilical scars |
| 24.5615 | -110.395 | 03/08/2017 | 439 | C | F | Neonate | 50.5 | 28.5 | Umbilical scars |
| 24.5616 | -110.395 | 03/08/2017 | 441 | C | M | Neonate | 55.0 | 32.5 | Umbilical scars |
| 24.5616 | -110.395 | 03/08/2017 | 354 | C | F | Neonate | 51.0 | 28.5 | Umbilical scars |
| 24.5616 | -110.395 | 03/08/2017 | 351 | C | F | Neonate | 50.0 | 29.5 | Umbilical scars |
| 24.5616 | -110.395 | 03/08/2017 | 440 | C | F | Neonate | 49.5 | 28.0 | Umbilical scars |
| 24.5619 | -110.395 | 07/10/2017 | 344 | C | M | Juvenile | 82.0 | 43.0 |  |
| 24.5619 | -110.395 | 07/10/2017 | 363 | R | M | Juvenile | 78.5 | 43.0 |  |
| 24.5619 | -110.395 | 07/10/2017 | 372 | C | F | Juvenile | 78.0 | 45.0 |  |
| 24.5619 | -110.395 | 07/10/2017 | 380 | C | F | Juvenile | 77.0 | 46.0 |  |
| 24.5619 | -110.395 | 07/10/2017 | 340 | C | M | Juvenile | 74.0 | 42.0 |  |
| 24.5619 | -110.395 | 07/10/2017 | 346 | R | M | Juvenile | 74.0 | 42.0 |  |
| 24.5619 | -110.395 | 07/10/2017 | 341 | C | F | Juvenile | 70.0 | 43.0 |  |
| 24.5619 | -110.395 | 07/10/2017 | 365 | C | F | Juvenile | 57.0 | 31.5 |  |
| 24.5619 | -110.395 | 07/10/2017 | 361 | C | F | Juvenile | 51.0 | 28.0 |  |
| 24.5597 | -110.396 | 08/10/2017 | 393 | C | F | Juvenile | 82.0 | 43.0 |  |
| 24.5597 | -110.396 | 08/10/2017 | 387 | C | M | Juvenile | 82.0 | 41.0 |  |
| 24.5597 | -110.396 | 08/10/2017 | 377 | C | M | Juvenile | 80.0 | 42.0 |  |
| 24.5597 | -110.396 | 08/10/2017 | 378 | C | M | Juvenile | 80.0 | 39.0 |  |
| 24.5597 | -110.396 | 08/10/2017 | 399 | C | M | Juvenile | 78.0 | 42.0 |  |
| 24.5597 | -110.396 | 08/10/2017 | 367 | C | M | Juvenile | 78.0 | 40.5 |  |
| 24.5597 | -110.396 | 08/10/2017 | 359 | C | M | Juvenile | 74.0 | 43.0 |  |
| 24.5597 | -110.396 | 08/10/2017 | 391 | C | F | Juvenile | 74.0 | 40.0 |  |
| 24.5597 | -110.396 | 08/10/2017 | 385 | C | M | Juvenile | 71.0 | 40.5 |  |
| 24.5597 | -110.396 | 08/10/2017 | 364 | C | F | Juvenile | 68.0 | 42.5 |  |
| 24.5597 | -110.396 | 08/10/2017 | 371 | C | M | Juvenile | 65.0 | 33.0 |  |
| 24.5597 | -110.396 | 08/10/2017 | 422 | C | M | Juvenile | 60.0 | 32.0 |  |
| 24.5597 | -110.396 | 08/10/2017 | 441 | R | M | Juvenile | 60.0 | 32.5 |  |
| 24.5597 | -110.396 | 08/10/2017 | 361 | R | F | Juvenile | 51.0 | 28.0 |  |
| 24.5597 | -110.396 | 08/10/2017 | 392 | C | M | Juvenile | 49.0 | 27.5 |  |
| 24.5607 | -110.396 | 03/12/2017 | 392 | R | M | Juvenile | 52.5 | 32.5 |  |
| 24.5607 | -110.396 | 03/12/2017 | 441 | R | M | Juvenile | 64.0 | 38.0 |  |
| 24.5607 | -110.396 | 03/12/2017 | 405 | C | M | Juvenile | 56.0 | 34.0 |  |
| 24.5612 | -110.397 | 05/04/2018 | 403 | C | F | Juvenile | 64.0 | 37.5 |  |
| 24.5604 | -110.406 | 05/04/2018 | 33580 | C | F | Adult | 110.0 | 70.0 | Distended cloaca and mating scars |
| 24.5604 | -110.406 | 05/04/2018 | 33581 | C | F | Adult | 110.0 | 66.5 | Distended cloaca and mating scars |
| 24.5604 | -110.406 | 05/04/2018 | 33588 | C | F | Adult | 103.5 | 65.0 | Distended cloaca and mating scars |
| 24.5604 | -110.406 | 05/04/2018 | 33831 | C | F | Adult | 102.0 | 63.0 | Distended cloaca and mating scars |
| 24.5579 | -110.397 | 06/04/2018 | 421 | C | F | Juvenile | 65.0 | 37.0 |  |
| 24.5579 | -110.397 | 06/04/2018 | 351 | R | F | Juvenile | 65.0 | 38.0 |  |
| 24.5579 | -110.399 | 07/04/2018 | 430 | C | F | Juvenile | 75.0 | 42.0 |  |
| 24.5579 | -110.399 | 07/04/2018 | 396 | C | M | Juvenile | 64.0 | 37.0 |  |
| 24.5578 | -110.399 | 07/04/2018 | 410 | C | F | Juvenile | 72.0 | 41.0 |  |
| 24.5574 | -110.399 | 07/04/2018 | 337 | C | F | Juvenile | 67.0 | 39.5 |  |
| 24.5558 | -110.405 | 04/06/2018 | 33593 | C | F | Adult | 102.0 | 66.0 |  |
| 24.5558 | -110.405 | 04/06/2018 | 33838 | C | M | Adult | 101.0 | 58.0 | Sperm on claspers |
| 24.5558 | -110.405 | 04/06/2018 | 33835 | C | M | Adult | 101.0 | 61.0 | Sperm on claspers |
| 24.5558 | -110.405 | 04/06/2018 | 33834 | C | M | Adult | 101.0 | 65.0 | Sperm on claspers |
| 24.5558 | -110.405 | 04/06/2018 | 33844 | C | M | Adult | 98.0 | 58.5 | Sperm on claspers |
| 24.5659 | -110.408 | 05/06/2018 | 33586 | C | M | Adult | 108.0 | 64.0 |  |
| 24.5659 | -110.408 | 05/06/2018 | 10 | C | F | Adult | 100.0 | 68.0 | Heavily pregnant |
| 24.5659 | -110.408 | 05/06/2018 | 33525 | C | M | Adult | 102.0 | 62.0 |  |
| 24.5659 | -110.408 | 05/06/2018 | 33837 | C | M | Adult | 105.0 | 61.0 |  |
| 24.5659 | -110.408 | 05/06/2018 | 33836 | C | M | Adult | 98.0 | 58.0 |  |
| 24.5659 | -110.408 | 05/06/2018 | 33833 | C | M | Adult | 98.0 | 61.0 |  |
| 24.5568 | -110.399 | 06/06/2018 | 400 | C | M | Juvenile | 73.0 | 39.0 |  |
| 24.5568 | -110.399 | 06/06/2018 | 412 | C | F | Juvenile | 87.0 | 52.5 |  |
| 24.5568 | -110.399 | 06/06/2018 | 406 | C | F | Juvenile | 80.0 | 47.5 |  |
| 24.5568 | -110.399 | 06/06/2018 | 401 | C | M | Juvenile | 69.0 | 39.0 |  |
