# Supplementary figures and images for: Description of first nursery area for a pygmy devil ray species (Mobula munkiana) in the Gulf of California, Mexico
Source: Sci Rep. 2021 Jan 8;11:132. doi: 10.1038/s41598-020-80506-8 (PMC7794486; doi:10.1038/s41598-020-80506-8)

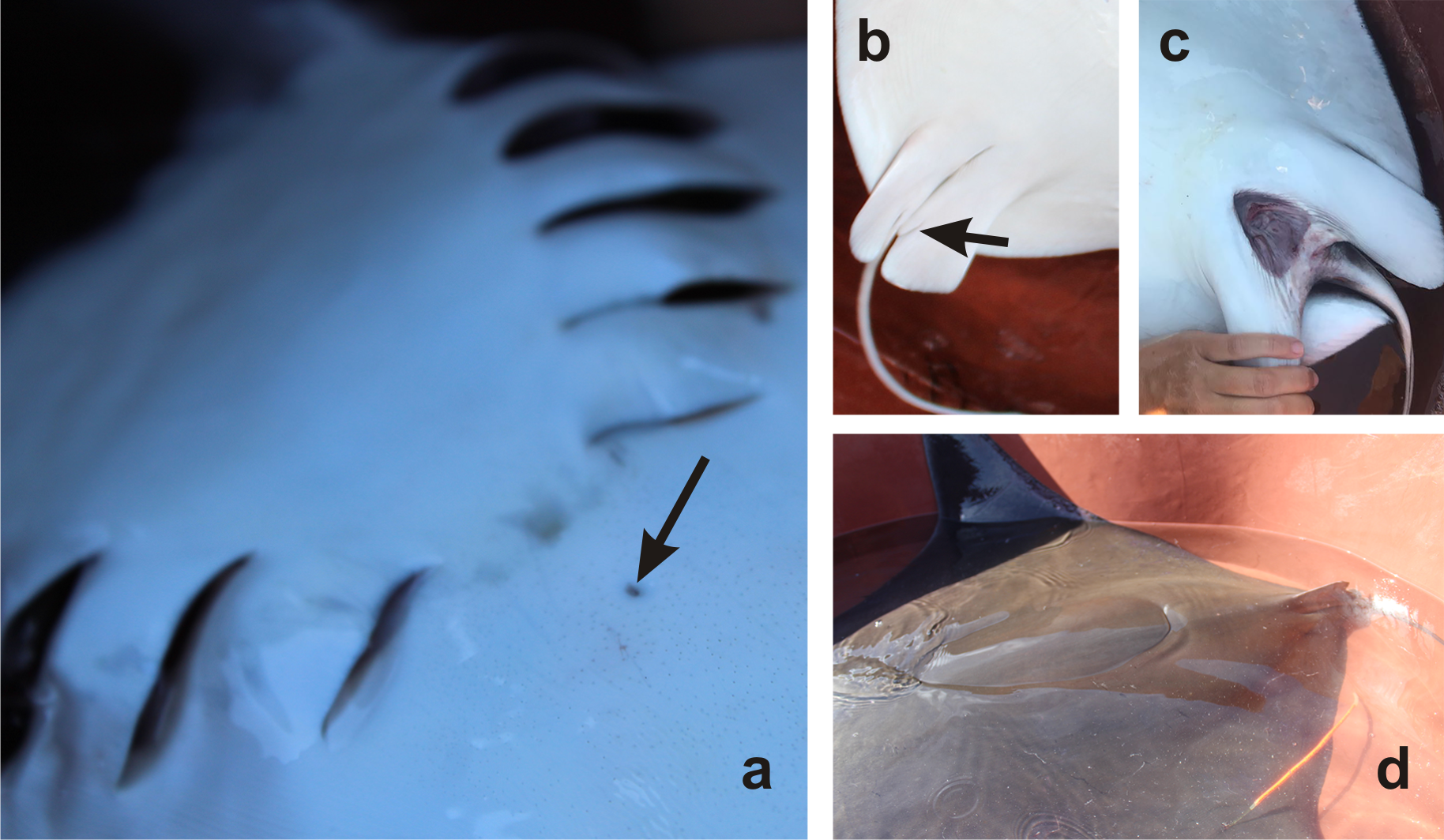

Supplement: Supplementary file 2 — Supplementary Figure S1. [file 41598_2020_80506_MOESM2_ESM.tif]
